# Rhinoplastic Operation for Epithelioma

**Published:** 1878-09-20

**Authors:** 


### RHINOPLASTIC OPERATION FOR EPITHELIOMA.

Prof. Hamilton, in a Clinical Lecture at Bellevue Hospital, says he perfarmed the second time on the same patient, a rhinoplastic operation for an epithelioma on the side of the nose, procuring a flap from the forehead and implanting it in the wound whence the diseased portion had been removed. From the time of the first operation to the second, an interval of twelve years had elapsed, during which time the patient had been free from the disease.

